# Spain is not different: teaching quantitative courses can also be hazardous to one’s career (at least in undergraduate courses)

**DOI:** 10.7717/peerj.13456

**Published:** 2022-05-31

**Authors:** Jose Luis Arroyo-Barrigüete, Antonio Obregón, José María Ortiz-Lozano, Antonio Rua-Vieites

**Affiliations:** 1 Quantitative Methods, Universidad Pontificia Comillas, Madrid, Madrid, Spain; 2 Public Law, Universidad Pontificia Comillas, Madrid, Madrid, Spain

**Keywords:** Student evaluation of teaching, Quantitative courses, Science education

## Abstract

Student evaluations of teaching (SETs) have become a widely used tool for assessing teaching in higher education. However, numerous investigations have shown that SETs are subject to multiple biases, one of which is particularly relevant, namely, the area of knowledge to which the subject belongs. This article aims to replicate the article by Uttl & Smibert (2017, https://doi.org/10.7717/peerj.3299) in a different educational context to verify whether the negative bias toward instructors who teach quantitative courses found by the authors in the US also appears in the Spanish university system. The study was conducted at the Business and Law School of the Universidad Pontificia Comillas, a private Spanish university, using two different samples. First, we analyzed undergraduate courses using a sample of 80,667 SETs in which 2,885 classes (defined as a single semester-long course taught by an individual instructor to a specific group of students), 488 instructors, and 322 different courses were evaluated over a time period of four academic years (2016/2017–2019/2020). Second, in the same period, 16,083 SETs corresponding to master’s degree courses were analyzed, which involved the study of 871 classes, 275 instructors, and 155 different courses. All the data included in the analysis were obtained from official university surveys developed by a team of professionals specialized in teaching quality responsible for ensuring the reliability of the information. At the degree level, the results show that despite the considerable cultural and temporal difference between the samples, the results are very similar to those obtained by Uttl & Smibert (2017, https://doi.org/10.7717/peerj.3299); *i.e*., professors teaching quantitative courses are far more likely to obtain worse SETs than instructors in other areas. There are hardly any differences at the master’s degree level, regardless of whether nearly 75% of master’s degree instructors also teach at the undergraduate level. This leads us to three different conclusions. (1) Evidence suggests that the reason for these differences is not due to faculty teaching quantitative courses being less effective than faculty teaching in some other fields. Our results indicate that the same instructor is evaluated very differently depending on whether he or she teaches at the undergraduate or master’s level. (2) It is essential to avoid comparisons of SETs between different areas of knowledge, at least at the undergraduate level. (3) A significant change in the use and interpretation of SETs is imperative, or its replacement by other evaluation mechanisms should be considered. If this does not occur, it is possible that in the future, there will be an adverse selection effect among professors of quantitative methods; *i.e*., only the worst professionals in quantitative methods will opt for teaching since the good professionals will prefer other jobs.

## Introduction

Student evaluations of teaching (SETs), even though they are widely used in universities around the world, continue to be surrounded by controversy as a result of the noninstructional biases that various studies have identified (see, for example, the recent review developed by Uttl, 2021). It is known that a large number of factors unrelated to teaching quality can bias the outcome of these evaluations. Some authors even go a step further, as in the case of Clayson (2022: 323), who proposes the likability hypothesis, suggesting that “the current evaluation system cannot validly measure anything other than what the students like and dislike”.

To mention just a few of the biases identified in the literature, there is strong evidence that instructors who give better grades obtain better SETs than those who are more demanding, although the learning achieved by students is higher in the second group. In a recent article, Berezvai, Lukáts & Molontay (2021: 793) concluded that “increasing the grade of a student by one will cause them to give approximately 0.2–0.4 higher evaluations for the instructor in the SET survey”. Indeed, several studies have reported that teachers who award higher grades receive better SETs than those who focus on the deeper learning that can be observed in later courses (Yunker & Yunker, 2003; Carrell & West, 2010; Kornell & Hausman, 2016). Braga, Paccagnella & Pellizzari (2014: 71) offered the following possible explanation: “teachers can either engage in real teaching or in teaching-to-the-test, the former requiring higher students’ effort than the latter. Teaching-to-the-test guarantees high grades in the current course but does not improve future outcomes”.

Related to the above, Felton, Mitchell & Stinson (2004) identified a student preference for course easiness using “Rate my professor” (RateMyProfessors.com) ratings. This result has been replicated using the same source by Felton et al. (2008), Rosen (2018), and Wallisch & Cachia (2019). Additionally, Arroyo-Barrigüete et al. (2021), using data from the Universidad Pontificia Comillas, concluded that instructors offering easy courses (low workload) tend to be rated highly. Uttl, Bell & Banks (2018) showed that class size also affects SETs, with a curvilinear relationship; *i.e*., SET ratings are the highest in the smallest classes, decline as the class size increases to 20–30 students, and then level off.

Many other biases have been identified, although it is true that in some cases, the evidence is contradictory or open to alternative interpretations. To give a few examples, Sanchez & Khan (2016) concluded that an instructor´s accent in online education does cause learners to rate the instructor as less effective, although this accent did not seem to affect the learning outcomes. However, students indeed reported greater comprehension difficulties with the accented instructor. Therefore, perhaps the bias detected is related to the additional effort needed, as comprehension scores decrease for nonnative (accented) instructors (Anderson-Hsieh & Koehler, 1988). This effect is also more noticeable in Sanchez & Khan’s experiment because the narrator was never presented in any visual capacity to the participants; thus, important comprehension signals such as gesturing (Goldin-Meadow, Kim & Singer, 1999) were lost.

Stonebraker & Stone (2015) found that instructor age negatively affects SETs, although the effect does not begin until the mid-forties. On the other hand, as the authors themselves indicate, the impact of age on SETs is small and can be offset by other factors, especially the physical appearance (the effect of age disappears for professors rated as “hot”) and how easy students consider them to be. The recent work by Tran & Do (2020) on a sample of 12,713 SETs of 124 courses concluded that age had no significant impact on SETs.

Finally, hundreds of studies investigated gender bias, but their conclusions are conflicting. This issue is not clear, as the gender effect could be an artifact of sample sizes, seniority, or field (see Uttl & Violo, 2021). As Uttl (2021: 246) indicates, “Gender differences could arise, be reduced, or even masked by a number of different factors”. Therefore, it is not completely clear whether there is indeed a gender bias or whether this is an artifact of other covariates.

Furthermore, we could mention many other potential noninstructional biases identified in various research studies, demonstrating that SETs depend on multiple factors unrelated to professor teaching effectiveness. Clayson (2009: 16) concluded that there exists a small average relationship between learning and SETs, and “the more objectively learning is measured, the less likely it is to be related to the evaluations”. The meta-analysis by Uttl, White & Gonzalez (2017) concluded that large sample-sized studies showed minimal correlation or even no correlation at all between SETs and learning, and consequently, SETs are not a valid measure of faculty teaching effectiveness. This conclusion is shared by Hornstein (2017) and Stroebe (2020), among others.

Nevertheless, the recent work by Uttl & Smibert (2017) highlights that a particularly relevant noninstructional bias, the subject area to which the course belongs, has likely been undervalued in many previous studies. Numerous articles have found a negative bias toward teachers who teach quantitative and/or STEM courses (Cashin, 1990; Beran & Violato, 2005; Centra, 2009; Uttl, White & Morin, 2013; Royal & Stockdale, 2015; DeFrain, 2016; Rosen, 2018; Arroyo-Barrigüete et al., 2021), but Uttl and Smibert claim that in several of these articles, the relevance of this bias is underestimated. They stated that parametric statistics are not appropriate due to several factors. First, the distributions of SETs are frequently negatively skewed due to ceiling effects. Second, to evaluate the effect of a variable on SETs, the method to be employed is the one that best reflects the effect of that variable on classifying professors as satisfactory/not satisfactory. Regardless of the percentage of the variance explained by that factor, the relevant metric is the risk of obtaining SETs below the cutoff value because this metric is the one that is frequently used to distinguish between good and bad performance. Therefore, the most appropriate effect size indices may be the relative risk ratio or odds ratios of professors passing the cutoff criteria instead of ds, rs, or R^2^. Consequently, using ds, rs or R^2^ results in underestimating the size of the effect, which does not seem as important as it is and can have a considerable impact on professors passing the minimum standard for satisfactory performance.

In a recent study by the same authors of the present research (Arroyo-Barrigüete et al., 2021), it was found that in the case of a business and law school (ICADE), the most relevant noninstructional variable was precisely whether the course belonged to the area of quantitative methods. However, the relative risk ratios of this area were not calculated, nor were their implications explored in depth; the focus of that work was on identifying the most relevant factors from among a total of 31 noninstructional variables identified in the literature, with a particular focus on the differences between areas, but without prioritizing anyone in particular. Starting from those results, that is, the notable negative bias identified toward quantitative courses, this article replicates the research of Uttl & Smibert (2017). There are three objectives. First, the aforementioned article uses a sample from a midsized US university. However, in this article, a sample from a midsized Spanish university is used. The second difference lies in the period under study since all the evaluations in the Uttl and Smibert study are prior to 2008, while the data used in this article correspond to 2016–2019. These two differences will allow us to assess whether the results of Uttl and Smibert can be extrapolated to other educational contexts and are also stable over time. Second, we intend to check whether these results are consistent when master’s degree courses are also considered. Therefore, we have added a sample of SETs at this educational level. Finally, in this article, we compare quantitative courses against every other area without focusing specifically on the area of languages to verify whether the differences found by Uttl and Smibert can be extrapolated to other fields of knowledge.

## Materials and Methods

This article received ethical approval from the Ethics Committee of the Universidad Pontificia Comillas (approval number 2021/94). The research is based on the results obtained by Arroyo-Barrigüete et al. (2021), going deeper into one of the findings of that work; in the case of a business and law school, the area of knowledge to which the course belongs induces the greatest bias. In that article, it was confirmed that once we control for the GPA effect (professors who give better grades obtain better SETs), workload (instructors offering easy courses tend to be rated highly), and other noninstructional biases, there is still a significant effect related to the type of course (area of knowledge). Furthermore, as Uttl & Smibert (2017) state, in addition to the percentage of variance explained by this variable, the marked negative skewness of the distributions means that the probabilities of obtaining poor SETs are very different depending on the type of course. Thus, starting from the results obtained in both investigations, this work aims to replicate the paper by Uttl & Smibert (2017).

The study was conducted at the Universidad Pontificia Comillas, a private Spanish university founded in 1890. The university comprises seven different schools, has over 13,000 students and approximately 1,700 lecturers, and offers 43 different undergraduate degrees and 33 master’s and doctoral programs. In this article, we have worked with the business and law school due to the aforementioned results obtained by Arroyo-Barrigüete et al. (2021). The sample for the undergraduate courses consists of 80,667 SETs and 2,885 classes in which 488 instructors and 322 different courses were evaluated over a time period of four academic years (2016/2017–2019/2020). The sample for the master courses consists of 16,083 SETs and 871 classes in which 275 instructors and 155 different courses were evaluated over the same period.

We used the class as the unit of analysis (a course taught by an instructor to a specific group of students). For each class, the average of the evaluations received by the instructor was calculated since it is the class average instead of individual student SETs that determines provosts’ assessments of university faculty.

The classification between quantitative and nonquantitative courses was made according to their content. All those with a strong mathematical, statistical or econometric content have been classified as quantitative. Therefore, an econometrics course has been classified as a quantitative course. Likewise, an experimental design course (with statistical content) or a financial mathematics course were also classified as quantitative. In the specific case of Universidad Pontificia Comillas, this classification is simple since all courses of this kind are taught by the Quantitative Methods Department.

A comparison of the density distributions for quantitative courses and the remaining courses was conducted, performing a k-sample Anderson–Darling test. We adopted a conservative alpha level of 0.005 to avoid false-positives (Benjamin et al., 2018). Additionally, in all cases, we calculated the risk ratio, which is the ratio of the probability of an outcome (SETs below the cutoff value used by the university to distinguish good and bad teachers) in an exposed group (quantitative courses) to the probability of an outcome in an unexposed group (nonquantitative courses). Mathematically, this is calculated as the result of dividing the cumulative incidence in the exposed group by the cumulative incidence in the unexposed group.

All the data included in this analysis were obtained from official university surveys developed by a team of experts in teaching quality. Table 1 shows the main statistics of the sample.

**Table 1:** Sample used in the study. SETs in a 1–10 scale.

		General	Law	Economics	Finance	Management	Languages	Marketing	Quant. methods	Int. relations
Undergraduate	# of subjects	13	113	24	31	32	44	10	22	33
	# of classes	348	1007	190	320	274	265	112	223	146
	# of instructors	63	165	29	60	69	28	36	30	28
	# of classes with a female/male instructor	123/225	450/557	87/103	124/196	102/172	153/112	78/34	107/116	32/114
	Average class size (SD)	46.80 (15.04)	54.73 (11.78)	50.17 (12.45)	47.23 (13.09)	46.77 (11.11)	34.72 (15.49)	45.12 (11.46)	54.7 (11.32)	53.29 (13.10)
	GPA: Mean (SD)/Median	8.00 (0.66)/8.08	6.98 (0.76)/6.94	7.23 (0.73)/7.17	6.90 (0.7)/6.85	7.50 (0.59)/7.51	7.41 (0.59)/7.43	7.60 (0.55)/7.59	6.49 (0.78)/6.38	7.67 (0.61)/7.63
	SET: Mean (SD)/Median	8.15 (1.16)/8.38	8.22 (1.09)/8.43	7.93 (1.43)/8.18	8.09 (1.20)/8.30	7.94 (1.19)/8.22	8.40 (0.90)/8.55	7.54 (1.51)/7.73	7.14 (1.27)/7.21	8.07 (1.15)/8.28
Master	# of subjects	17	13	5	38	46	–	22	9	5
	# of classes	85	44	36	319	232	–	47	83	25
	# of instructors	32	26	13	70	101	–	24	19	18
	# of classes with a female/male instructor	8/77	10/34	16/20	104/215	62/170	–	20/27	19/64	5/20
	Average class size (SD)	22.61 (8.13)	22.95 (9.12)	19.056 (6.56)	23.06 (9.96)	21.63 (8.48)	–	18.85 (7.44)	22.13 (10.6)	18.6 (6.88)
	GPA: Mean (SD)/Median	7.81 (0.99)/7.94	7.52 (0.84)/7.49	7.65 (1.11)/7.93	7.53 (0.68)/7.52	7.66 (1.01)/7.835	–	7.90 (0.76)/8.04	7.44 (0.63)/7.5	8.22 (0.58)/8.15
	SET: Mean (SD)/Median	8.09 (1.47)/8.63	8.16 (0.92)/8.09	8.77 (0.91)/9.00	8.49 (1.17)/8.87	8.09 (1.27)/8.27	–	8.28 (1.26)/8.63	8.43 (0.91)/8.69	8.65 (1.05)/8.90

Although the difference is minimal, it should be noted that the sum of professors by area does not exactly coincide with the number of professors included in the sample (275 – master courses/488 – undergraduate courses), since a few instructors teach in several categories (typically in courses in their area of knowledge and in the area of “general contents”).

The analyses were conducted in R (R Core Team, 2020), a software environment for statistical computing and graphics, using several packages to elaborate the code: gridExtra (Auguie, 2017), readxl (Wickham & Bryan, 2019), fmsb (Nakazawa, 2019), KSamples (Scholz & Zhu, 2019), RcmdrMisc (Fox, 2020), dplyr (Wickham et al., 2020) and ggplot2 (Wickham, 2016).

## Results

**Figure 1:**
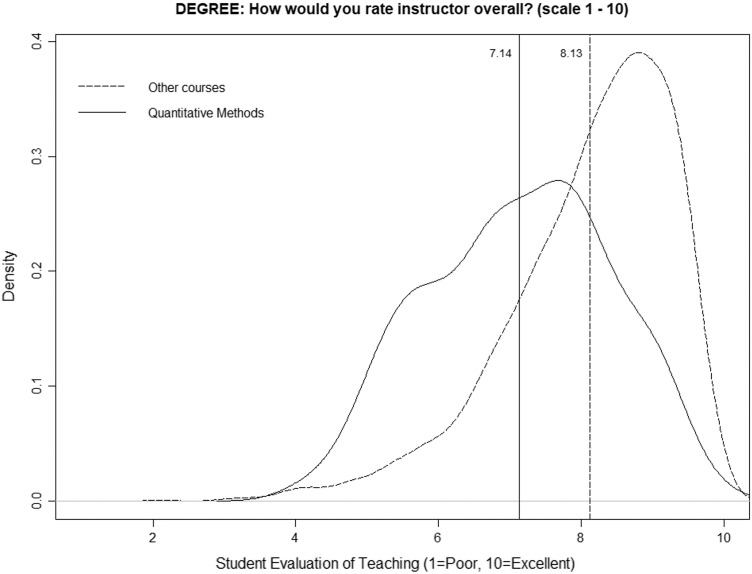
Distributions of mean ratings for quantitative and nonquantitative courses (degree programs).

Figure 1 shows the smoothed density distribution of the mean ratings for quantitative courses and for nonquantitative courses (degree programs) on a scale from 1 to 10. The figure highlights that the distributions are considerably different among quantitative and nonquantitative courses, with the difference being an average value of one point (vertical lines), *i.e*., 7.14 for quantitative courses and 8.13 for nonquantitative courses. Following Uttl & Smibert (2017), the figure was generated using the R function “density” with a smoothing kernel set to “Gaussian.” The *p*-value obtained in the k-sample Anderson–Darling test is very low (1.88E−37), so we can state that the two distributions are different.

**Figure 2:**
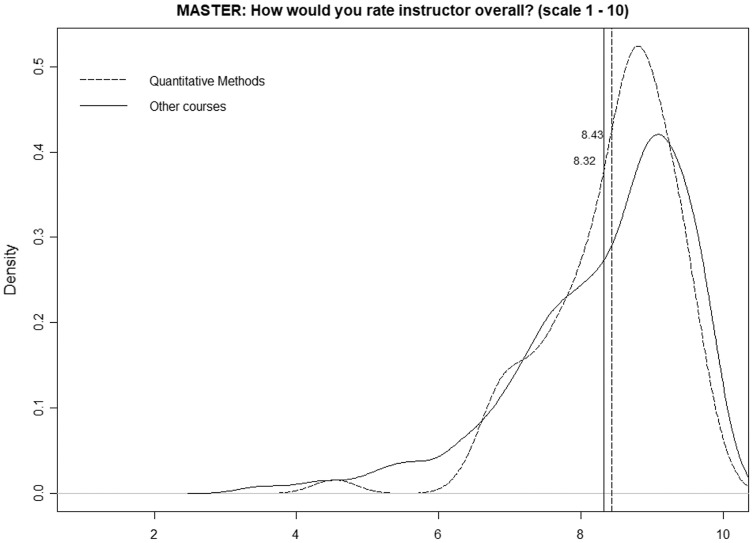
Distributions of mean ratings for quantitative and nonquantitative courses (master’s programs).

Figure 2 is identical but only considers the master’s programs, and we can observe a very different scenario, namely, both distributions and average values are quite similar (8.43 for quantitative courses and 8.34 for nonquantitative courses). The *p*-value obtained in the k-sample Anderson–Darling test is relatively high (0.099), so we cannot state that the two distributions are different.

**Figure 3:**
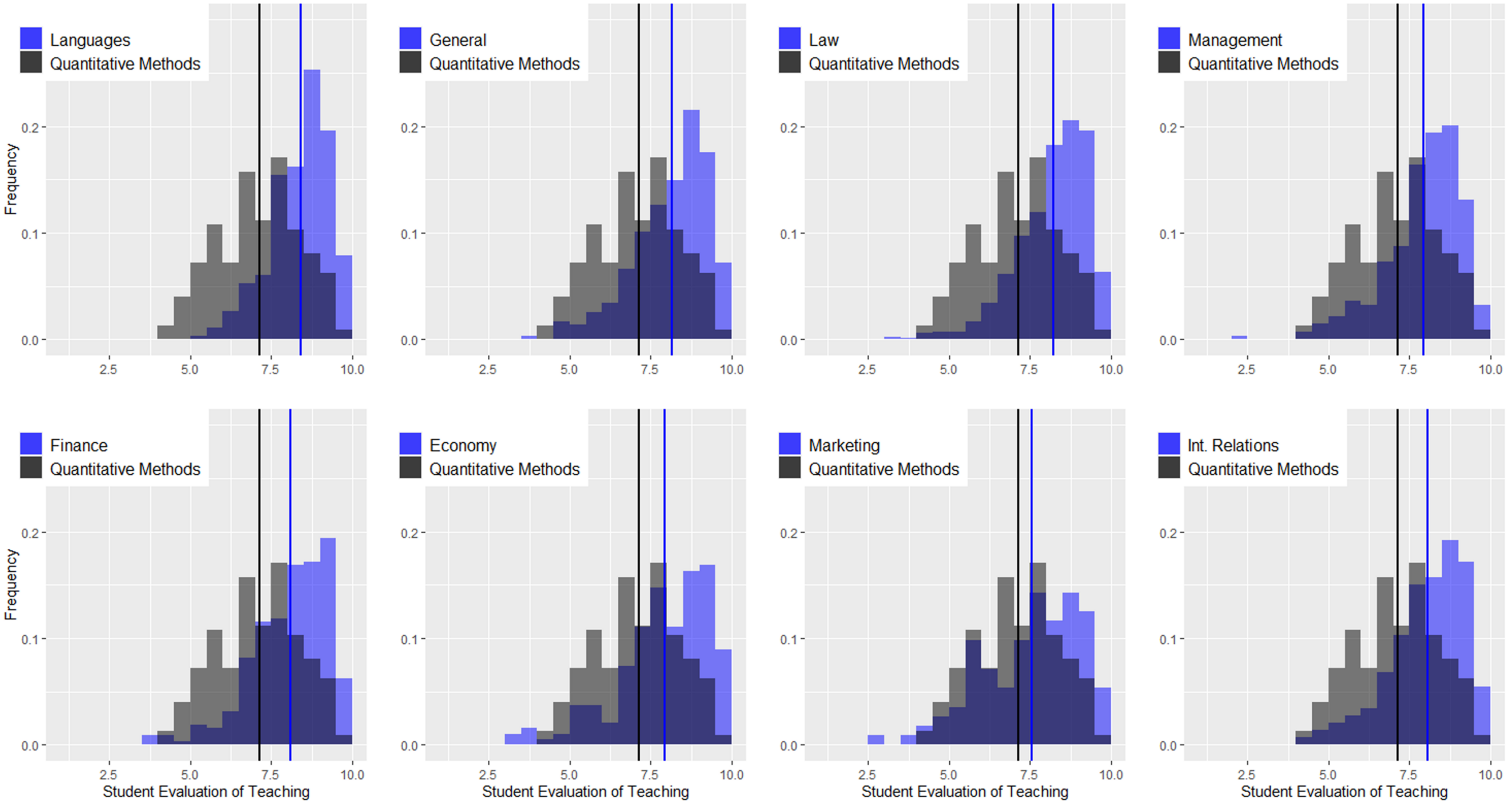
Density distributions for quantitative courses and the remaining courses at the undergraduate level.

**Figure 4:**
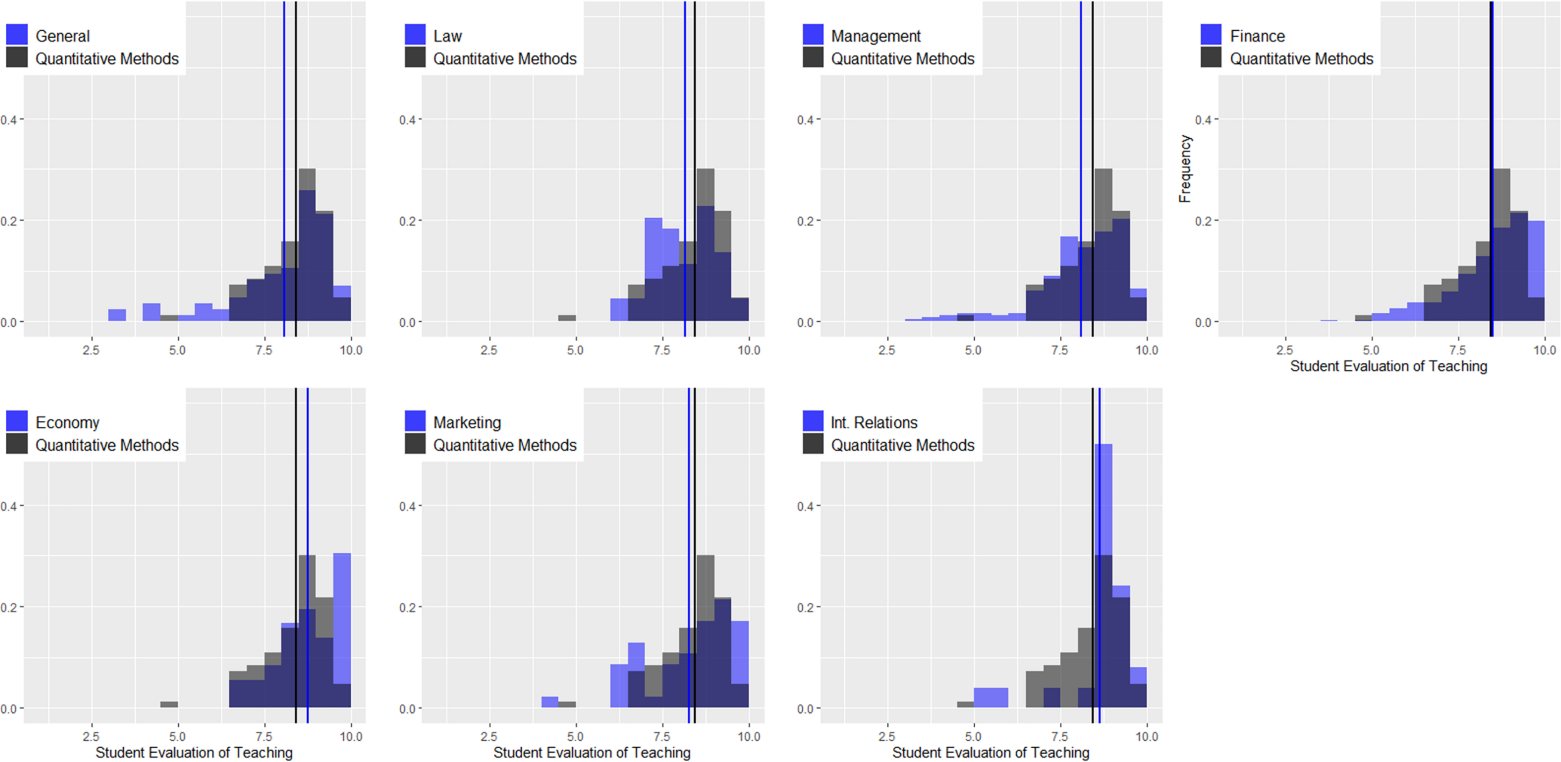
Density distributions for quantitative courses and the remaining courses at the master level.

Figure 3 shows the density distributions for quantitative courses and the remaining courses at the undergraduate level (languages, general contents, law, management, finance, economics, marketing, and international relations). The vertical lines indicate the average values. There are several conclusions related to this figure. (1) Regardless of the area, the instructors of quantitative courses obtain worse evaluations than those of other areas. In some cases, the differences are considerable, as in the case of languages and law, and in others, they are not as pronounced, as in the case of marketing. The K-sample Anderson–Darling test shows statistically significant differences for all comparisons (see Table 2). (2) In the remaining areas, the distributions of the ratings are negatively skewed, and the only area in which this does not happen is precisely in quantitative methods. (3) Combining both effects, we can conclude that regardless of the cutoff criterion used by the university to distinguish good and bad teachers, instructors in the area of quantitative methods will always be disadvantaged in comparative terms. Figure 4 is identical but only considers the master’s programs, and again, we can observe a very different scenario. (1) The instructors of quantitative courses obtain similar or even better evaluations than instructors of other areas. The K-sample Anderson–Darling test shows no statistically significant differences for any comparison (see Table 2). (2) Evaluations in quantitative methods present negative asymmetry, and (3) both factors lead to instructors in this area not being disadvantaged by the application of equal cutoff criteria for all areas.

**Table 2:** K-sample Anderson–Darling test comparing the distribution in quantitative courses *vs*. other courses.

		General	Law	Economics	Finance	Management	Languages	Marketing	Int. relations
Undergraduate	Test statistic	54.76	92.63	27.1	48.42	34.05	79.68	6.60	30.72
	*P*-Value	1.32E−23	2.20E−39	4.42E−12	5.82E−21	5.64E−15	5.48E−34	9.21E−04	1.38E−13
Master	Test statistic	0.46	1.34	2.89	3.60	1.96	–	0.92	2.00
	*P*-Value	0.216	0.090	0.022	0.012	0.050	–	0.136	0.048

**Figure 5:**
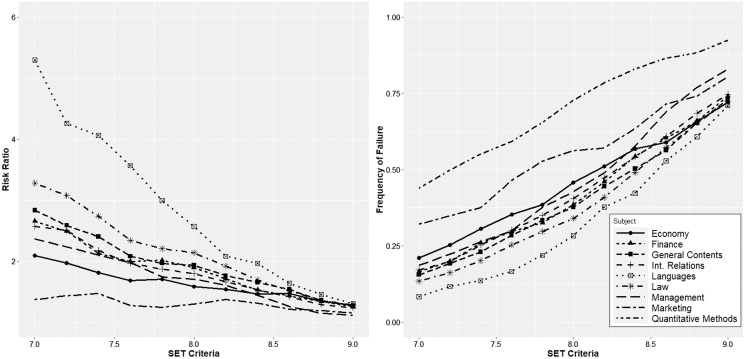
Relative risk ratio for different cutoff values in the undergraduate programs, and percentage of instructors who would fall below each cutoff value.

However, regardless of the shape of the distributions, universities typically use a certain cutoff value to distinguish professors with good or bad performance. In the Spanish system, one of the usual forms of calculating SETs is on a scale of 1 to 10. It is an easy-to-understand scale that replicates the one used to evaluate students. The cutoff value is usually approximately 7 or 8; teachers with SETs below this value should improve their teaching. Therefore, in Fig. 5, we calculated the relative risk ratio for different cutoff values in the undergraduate programs using an interval from 7 to 9, covering all possible values within reason. The relative risk ratios were calculated in all cases compared to quantitative methods courses. We also calculated the percentage of instructors who would fall below each cutoff value. The numerical data can be found in Table 3. The results confirm that regardless of the cutoff value selected, quantitative methods instructors are always disadvantaged, dramatically in some cases (as is the case when compared with languages). Moreover, the problem is not solved by lowering the cutoff value since, due to the shape of the distributions, such a measure will decrease the percentage of professors below the threshold value but will increase the relative risk ratio with the remaining courses. Focusing on a specific case, for a cutoff value of 8, we find that 72.6% of the professors of quantitative methods would be included in the “poor performance” category. In comparison, the percentages are substantially lower in the remaining areas: 28.3% in languages, 34.0% in law, 37.6% in general contents, 38.4% in finance, 40.4% in international relations, 42.7% in management, 45.8% in economics and 56.3% in marketing, implying that the relative risk ratio is much higher with the resulting consequences for career development.

**Table 3:** Risk ratio and % of instructors below the threshold for different cutoff values (undergraduate courses).

Cut-off criterion	Quantitative method fail (%)	Languages fail (%)	RR	*p*-value	General cont. fail (%)	RR	*p*-value	Law fail (%)	RR	*p*-value	Management fail (%)	RR	*p*-value
6	22.9	1.1	20.2	3E−14	5.7	4.0	2E−09	3.8	6.1	0E+00	8.4	2.7	7E−06
6.2	25.6	2.3	11.3	2E−14	7.2	3.6	1E−09	4.6	5.6	0E+00	9.1	2.8	9E−07
6.4	28.7	3.8	7.6	2E−14	8.0	3.6	6E−11	6.5	4.4	0E+00	10.6	2.7	3E−07
6.6	33.2	4.2	8.0	0E+00	10.1	3.3	7E−12	7.9	4.2	0E+00	13.9	2.4	3E−07
6.8	39.9	6.0	6.6	0E+00	12.4	3.2	3E−14	10.4	3.8	0E+00	16.1	2.5	2E−09
7	43.9	8.3	5.3	0E+00	15.5	2.8	7E−14	13.4	3.3	0E+00	18.6	2.4	9E−10
7.2	49.8	11.7	4.3	0E+00	19.3	2.6	2E−14	16.2	3.1	0E+00	22.3	2.2	1E−10
7.4	55.2	13.6	4.1	0E+00	23.0	2.4	5E−15	20.2	2.7	0E+00	26.3	2.1	6E−11
7.6	59.2	16.6	3.6	0E+00	28.4	2.1	3E−13	25.3	2.3	0E+00	29.9	2.0	6E−11
7.8	65.5	21.9	3.0	0E+00	33.3	2.0	6E−14	29.7	2.2	0E+00	37.6	1.7	7E−10
8	72.6	28.3	2.6	0E+00	37.6	1.9	4E−16	34.0	2.1	0E+00	42.7	1.7	2E−11
8.2	78.5	37.7	2.1	0E+00	44.5	1.8	1E−15	40.9	1.9	0E+00	48.9	1.6	1E−11
8.4	83.0	42.3	2.0	0E+00	50.3	1.6	3E−15	49.1	1.7	0E+00	57.7	1.4	1E−09
8.6	86.5	52.8	1.6	2E−15	56.3	1.5	4E−14	57.2	1.5	2E−16	69.0	1.3	4E−06
8.8	88.3	60.8	1.5	7E−12	65.2	1.4	7E−10	65.3	1.4	2E−11	77.0	1.1	1E−03
9	92.4	70.9	1.3	2E−09	73.0	1.3	1E−08	72.2	1.3	2E−10	82.8	1.1	2E−03
**Cut-off criterion**	**Quantitative method fail (%)**	**Finance fail (%)**	**RR**	***p*-value**	**Economics fail (%)**	**RR**	***p*-value**	**Marketing fail (%)**	**RR**	***p*-value**	**I. relations fail (%)**	**RR**	***p*-value**
6	22.9	5.6	4.1	3E−09	10.5	2.2	9E−04	19.6	1.2	5E−01	6.8	3.3	5E−05
6.2	25.6	6.6	3.9	6E−10	12.1	2.1	6E−04	22.3	1.1	5E−01	7.5	3.4	1E−05
6.4	28.7	7.8	3.7	1E−10	13.2	2.2	1E−04	26.8	1.1	7E−01	8.2	3.5	2E−06
6.6	33.2	10.9	3.0	2E−10	14.7	2.3	2E−05	26.8	1.2	2E−01	11.6	2.8	3E−06
6.8	39.9	13.8	2.9	3E−12	17.9	2.2	1E−06	28.6	1.4	4E−02	13.0	3.1	3E−08
7	43.9	16.6	2.7	3E−12	21.1	2.1	9E−07	32.1	1.4	4E−02	17.1	2.6	9E−08
7.2	49.8	20.0	2.5	3E−13	25.3	2.0	3E−07	34.8	1.4	1E−02	19.9	2.5	7E−09
7.4	55.2	25.9	2.1	5E−12	30.5	1.8	5E−07	37.5	1.5	2E−03	25.3	2.2	2E−08
7.6	59.2	29.7	2.0	7E−12	35.3	1.7	1E−06	46.4	1.3	3E−02	30.1	2.0	5E−08
7.8	65.5	32.5	2.0	4E−14	38.4	1.7	4E−08	52.7	1.2	2E−02	34.9	1.9	9E−09
8	72.6	38.4	1.9	4E−15	45.8	1.6	3E−08	56.3	1.3	3E−03	40.4	1.8	7E−10
8.2	78.5	45.9	1.7	3E−14	51.1	1.5	5E−09	57.1	1.4	5E−05	47.3	1.7	6E−10
8.4	83.0	54.4	1.5	5E−12	56.8	1.5	6E−09	63.4	1.3	7E−05	54.1	1.5	2E−09
8.6	86.5	60.3	1.4	3E−11	58.9	1.5	2E−10	71.4	1.2	8E−04	61.0	1.4	2E−08
8.8	88.3	65.9	1.3	3E−09	65.8	1.3	4E−08	74.1	1.2	9E−04	68.5	1.3	3E−06
9	92.4	74.1	1.2	6E−08	72.1	1.3	5E−08	80.4	1.1	1E−03	74.7	1.2	3E−06

In the case of the master’s programs, again, the situation is different. The same analysis shows that most relative risk ratios are not significant. Only for some cutoff values are they significant, and these values are different depending on the areas (see Table 4). Therefore, although there are statistically significant differences for some values, sometimes the differences favor and sometimes disfavor the quantitative methods courses without establishing a single criterion. The conclusion, therefore, is that in master’s programs, quantitative methods instructors are neither disadvantaged nor favored with respect to other areas.

**Table 4:** Risk ratio and % of instructors below the threshold for different cutoff values (master courses).

Cut-off criterion	Quantitative method fail (%)	General cont. fail (%)	RR	*p*-value	Law fail (%)	RR	*p*-value	Management fail (%)	RR	*p*-value	Finance fail (%)	RR	*p*-value
6	1.2	10.6	0.1	1E−02	0.0	Inf	5E−01	7.3	0.2	4E−02	4.4	0.3	2E−01
6.2	1.2	11.8	0.1	6E−03	2.3	0.5	6E−01	8.2	0.1	3E−02	5.3	0.2	1E−01
6.4	1.2	12.9	0.1	3E−03	2.3	0.5	6E−01	9.1	0.1	2E−02	7.2	0.2	4E−02
6.6	2.4	14.1	0.2	6E−03	4.5	0.5	5E−01	9.5	0.3	4E−02	8.8	0.3	5E−02
6.8	2.4	15.3	0.2	4E−03	4.5	0.5	5E−01	11.2	0.2	2E−02	9.7	0.2	3E−02
7	8.4	16.5	0.5	1E−01	6.8	1.2	7E−01	13.4	0.6	2E−01	11.6	0.7	4E−01
7.2	12.0	22.4	0.5	8E−02	15.9	0.8	5E−01	16.8	0.7	3E−01	14.7	0.8	5E−01
7.4	13.3	23.5	0.6	9E−02	25.0	0.5	1E−01	21.1	0.6	1E−01	16.3	0.8	5E−01
7.6	16.9	28.2	0.6	8E−02	34.1	0.5	3E−02	26.7	0.6	7E−02	20.4	0.8	5E−01
7.8	20.5	30.6	0.7	1E−01	40.9	0.5	1E−02	34.9	0.6	1E−02	24.8	0.8	4E−01
8	25.3	34.1	0.7	2E−01	45.5	0.6	2E−02	40.1	0.6	2E−02	26.6	0.9	8E−01
8.2	32.5	37.6	0.9	5E−01	56.8	0.6	8E−03	47.0	0.7	2E−02	32.9	1.0	9E−01
8.4	38.6	44.7	0.9	4E−01	59.1	0.7	3E−02	52.2	0.7	3E−02	38.6	1.0	1E+00
8.6	45.8	48.2	0.9	8E−01	59.1	0.8	2E−01	57.8	0.8	6E−02	42.6	1.1	6E−01
8.8	57.8	60.0	1.0	8E−01	63.6	0.9	5E−01	65.5	0.9	2E−01	48.6	1.2	1E−01
9	72.3	67.1	1.1	5E−01	77.3	0.9	5E−01	72.0	1.0	1E+00	57.1	1.3	1E−02
**Cut-off criterion**	**Quantitative method fail (%)**	**Economics fail (%)**	**RR**	***p*-value**	**Marketing fail (%)**	**RR**	***p*-value**	**I. Relations fail (%)**	**RR**	***p*-value**			
6	1.2	0.0	Inf	5E−01	2.1	0.6	7E−01	8.0	0.2	7E−02			
6.2	1.2	0.0	Inf	5E−01	2.1	0.6	7E−01	8.0	0.2	7E−02			
6.4	1.2	0.0	Inf	5E−01	8.5	0.1	4E−02	8.0	0.2	7E−02			
6.6	2.4	0.0	Inf	3E−01	12.8	0.2	2E−02	8.0	0.3	2E−01			
6.8	2.4	2.8	0.9	9E−01	14.9	0.2	7E−03	8.0	0.3	2E−01			
7	8.4	5.6	1.5	6E−01	21.3	0.4	4E−02	8.0	1.1	9E−01			
7.2	12.0	8.3	1.4	6E−01	23.4	0.5	9E−02	8.0	1.5	6E−01			
7.4	13.3	11.1	1.2	7E−01	25.5	0.5	8E−02	12.0	1.1	9E−01			
7.6	16.9	11.1	1.5	4E−01	29.8	0.6	9E−02	12.0	1.4	6E−01			
7.8	20.5	16.7	1.2	6E−01	31.9	0.6	1E−01	12.0	1.7	3E−01			
8	25.3	16.7	1.5	3E−01	34.0	0.7	3E−01	12.0	2.1	2E−01			
8.2	32.5	22.2	1.5	3E−01	38.3	0.8	5E−01	12.0	2.7	5E−02			
8.4	38.6	33.3	1.2	6E−01	44.7	0.9	5E−01	12.0	3.2	1E−02			
8.6	45.8	36.1	1.3	3E−01	44.7	1.0	9E−01	16.0	2.9	8E−03			
8.8	57.8	44.4	1.3	2E−01	55.3	1.0	8E−01	36.0	1.6	6E−02			
9	72.3	47.2	1.5	9E−03	61.7	1.2	2E−01	56.0	1.3	1E−01			

## Discussion

First, it is very interesting to confirm that the results obtained by Uttl & Smibert (2017) for the comparison between math and English undergraduate courses are very similar to those obtained in this article. Table 5 uses the same cutoff criteria as those authors for comparative purposes. The similarities are remarkable, which is of particular interest because they come from completely different educational contexts (USA *vs*. Spain) and very different time periods (SETs prior to 2008 *vs*. SETs from 2016–2019). Therefore, the first conclusion is that the results obtained by Uttl & Smibert (2017) are remarkably robust even when the sample considered is substantially modified.

**Table 5:** Comparison of Uttl & Smibert (2017) results with those obtained in this article.

	Uttl & Smibert (2017)			Own analysis		
	Math fail (%)	English fail	Math *vs*. English RR of failure	Quantitative fail (%)	Languages fail	Quantitative *vs*. Languages RR of failure
Norm-referenced						
Mean	78.6	28.7	2.74	74.9	30.9	2.42
Mean minus 1 SD	42.0	6.9	6.05	39.9	6.0	6.84
Mean minus 2 SD	16.1	1.4	11.59	14.7	0.4	39.21

**Figure 6:**
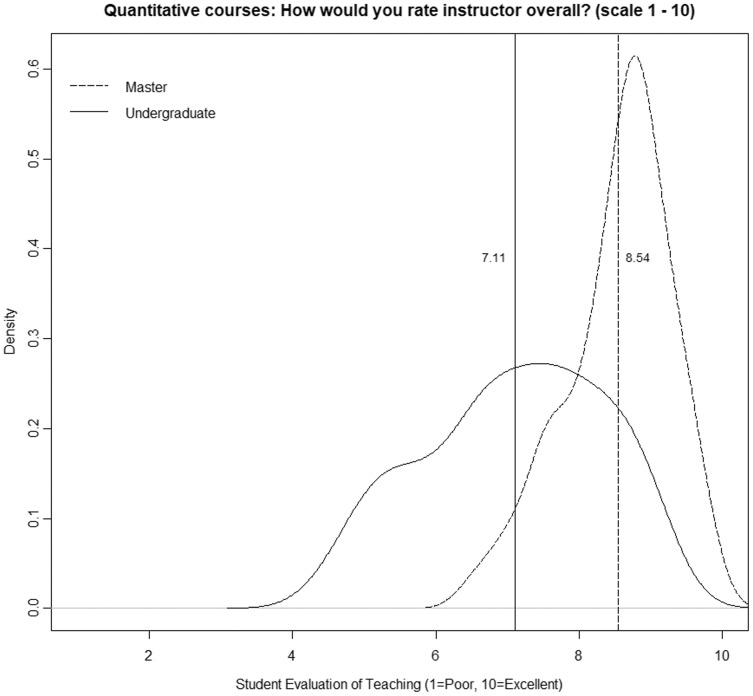
Quantitative courses: how would you rate instructor overall? (scale 1–10).

In this sense, it has been confirmed that in undergraduate courses, the area of knowledge induces a considerable bias in SETs (Cashin, 1990; Beran & Violato, 2005; Centra, 2009; Uttl, White & Morin, 2013; Royal & Stockdale, 2015; Uttl & Smibert, 2017; Arroyo-Barrigüete et al., 2021). Furthermore, it is also clear that this bias has relevance, regardless of the percentage of the variance explained by that factor. Instructors of quantitative methods have a much higher risk of obtaining SETs below the cutoff value than instructors of other areas. Moreover, this conclusion is the same for any cutoff value and for any other area. Simply put, these professors suffer a penalty for the mere fact that they are teaching quantitative methods. Of course, the differences are more significant for some areas (languages or law) than for others (economics or marketing). However, in all cases, regardless of the cutoff value chosen by the university to differentiate between good and bad performance, quantitative methods instructors will be at much greater risk of being included in the “poor performance” category. It could be argued that to the extent that crude analysis has been conducted without controlling for the numerous confounding factors that affect SETs, the results may be distorted. Moreover, as some authors have suggested, perhaps the problem is precisely that teachers of quantitative courses are worse than teachers of other areas, so their evaluations do not respond to noninstructional biases but to this fact. Cashin (1990: 119) suggests as a possible explanation that “some academic fields are poorly taught. Many of the low-rated fields are those in which institutions must pay very high salaries even to compete modestly with business and industry. Perhaps the faculty teaching those courses are less effective as a group than faculty in some other fields. It costs far less to hire an outstanding teacher in English than it does to hire an outstanding teacher in computer science, accounting, or engineering”. Our analysis suggests that this is not the reason. To explore this possibility, we selected only those professors who simultaneously teach bachelor’s and master’s degree courses, focusing on quantitative courses. Table 6 shows paired data for each instructor. As can be seen, in all cases and without exception, there is an improvement of SETs in master, ranging from 3.6% to 57.7%. In addition, the percentage of courses with a SET below the cutoff value of 8 also varies significantly in almost all cases. Figure 6 shows the smoothed density distribution of the mean ratings for quantitative courses at the undergraduate level (112 classes) and for the master’s level (65 classes), considering only those instructors. The figure was generated using the R function “density” with a smoothing kernel set to “Gaussian”. The figure highlights that the distributions are considerably different (the *p*-value obtained in the k-sample Anderson–Darling test is 1.6 E−15), with the difference being an average value of 1.4 points (vertical lines). Discrepancies are considerable, although we are analyzing the same group of instructors in both cases. Certainly, given that class size in master courses is significantly smaller, it is possible that part of the difference is due to this bias (see Uttl, Bell & Banks, 2018). However, the differences are too large for that to be the explanatory factor, especially when areas such as “management”, “general contents,” and “law” do not see the same effect.

**Table 6:** Professors of quantitative methods who simultaneously teach bachelor’s and master’s degree courses.

	# Courses		SETs (mean)			% Fail (cutoff value of 8)	
	Undergraduate	Master	Undergraduate	Master	Difference (%)	Undergraduate (%)	Master (%)
Professor 1	3	5	7.37	8.83	19.8	100	0
Professor 2	4	11	8.34	8.64	3.6	25	18
Professor 3	6	4	6.40	7.89	23.3	100	50
Professor 4	7	13	8.03	8.56	6.7	14	23
Professor 5	10	4	8.11	8.84	9.1	40	25
Professor 6	11	11	7.60	8.36	10.1	73	27
Professor 7	11	1	6.13	6.82	11.2	100	100
Professor 8	13	5	6.65	8.70	30.8	92	20
Professor 9	14	8	8.48	8.79	3.7	14	0
Professor 10	14	2	5.51	8.69	57.7	100	0
Professor 11	19	1	6.91	7.76	12.2	100	100

In other words, the same professor is evaluated differently depending on whether he or she teaches undergraduate or master’s courses. This evidence suggests that the marked negative bias toward quantitative courses at the undergraduate level is not due to the deficits of the instructors themselves, since such deficits would also be seen at the master’s level, which is not the case. However, other possible explanations need to be considered. First, it is possible that instructors are more motivated or put more effort into master teaching than into undergraduate teaching. In the specific case of Universidad Pontificia Comillas, this is unlikely to be the case. On the one hand, let us consider the instructor who wants to teach to SETs (to get the highest SETs possible). For this instructor profile, it makes no sense to differentiate between undergraduate and master courses because the evaluation of instructors does not distinguish between the two levels; an instructor who aspires to be promoted must obtain good SETs in both undergraduate courses and master courses. Focusing efforts exclusively on master courses would lead to a negative evaluation in undergraduate courses and, consequently, a loss of promotion. Perhaps there is a little extra motivation for part-time teachers, as they are teaching potential future colleagues. However, even in these cases, if their evaluations in undergraduate courses are low, they will probably be dismissed. On the other hand, let us consider the instructor who enjoys teaching and SETs are not his or her priority. Most likely, this instructor will enjoy teaching students who are there to learn and may invest more effort teaching master-level students who are more likely to be interested in the subject. We cannot rule out this possibility, but there are some indications that it is not a common occurrence. The first is that the improvement in master courses does not occur in all subjects, and in “management”, “general content,” and “law,” the SETs are virtually identical. The second is that if this type of behavior is detected at the university, the instructor will receive a warning for not paying enough attention to the undergraduate courses. At Universidad Pontificia Comillas, undergraduate programs are one of the cornerstones of its educational strategy, with a much higher volume of students than in master’s or PhD programs. They are therefore a priority, and instructors would not be allowed to neglect them to devote more effort to master’s courses.

A second possibility is that instructors opt to use their better teaching assistants for the master courses. Actually, this is not possible in the Universidad Pontificia Comillas since there are no teaching assistants. In fact, this role is not widespread in the Spanish university system. Therefore, the only possible explanation is that the student population is very different at the master level than at the undergraduate level. Students who choose a master’s degree with quantitative content are genuinely interested in it, as there are many other alternatives available that are free of such content. In contrast, undergraduate business administration students are forced to take quantitative courses. Therefore, the question that arises is whether the effect detected in quantitative courses is a problem of mandatory *vs*. elective classes. However, in the Spanish university system, nearly 80–90% of all undergraduate courses are mandatory. In the sample of undergraduate courses considered in this article, 223 classes (7.7%) correspond to elective courses, and 2,662 classes (92.3%) correspond to mandatory courses. Nevertheless, the enormous negative bias toward quantitative courses has not been detected in other disciplines where almost all courses are also mandatory.

In our opinion, a possible explanation for this phenomenon can be found in motivational factors; as mentioned, the student population is very different at the master level than at the undergraduate level. A student who wishes to pursue a degree in business administration is more likely to be interested in finance or management courses than in mathematics or statistics. These quantitative courses are compulsory to obtain a diploma, so the student must study them, but their approach to the course will probably not be the most positive. This effect was detected by Uttl, White & Morin (2013) among undergraduate psychology students; out of 340 participants, fewer than 10 were very interested in taking a quantitative course, but 159 were very interested in taking the Introduction to the Psychology of Abnormal Behavior. In fact, the mean interest in statistics courses was nearly 6 standard deviations below the mean interest in nonquantitative courses. In other words, the Spanish university system forces undergraduate business administration students to take quantitative courses that are probably very unattractive to them. In addition, there is no possibility of avoiding these courses. However, master’s degrees allow a high level of specialization so that it is possible to choose the path that best suits each student’s interests. Students who choose a master’s degree with quantitative content are truly interested in it, as there are many other alternatives available that are free of such content. Hence, their approach to the courses, and therefore the SETs, are more positive.

We must be aware that the mathematical level in Spain is low compared to that in other countries. The latest PISA report, which measures the mathematical literacy of 15-year-old students, indicates that the mathematical level in Spain is below the OECD average (OECD, 2018). This leads to a clear disinterest in this subject. A recent study in Spain (Pedrosa, 2020) on a sample of 1,293 students with various degrees (food and agricultural engineering, biology, food science and technology, childhood education, computer engineering, primary education and tourism) concluded that “Students do not like mathematics, do not enjoy using it, do not enjoy talking about it, and do not feel motivated to study it, so they would not take mathematics courses voluntarily, nor would they want a job in which they would have to use it” (pp. 174–175). Interest in taking courses has an important effect on SETs, as has been shown in several studies (Hoyt & Lee, 2002; Rampichini, Grilli & Petrucci, 2004; Wolbring, 2012; La Rocca et al., 2017; Sulis, Porcu & Capursi, 2019).

The perceived usefulness of the course could also play a relevant role; it is quite possible that master’s students perceive quantitative courses as fundamental for career development. Undergraduate students who also tend to take these courses in their first years and are therefore still far from the labor market find it more difficult to perceive their usefulness in terms of professional development. Finally, we can hypothesize a possible “gratification effect”; *i.e*., perhaps quantitative courses do not provide gratification to business administration students due to several factors. First, these courses are perceived as a continuation of high school, so the “discovery factor” is less than that in other completely new subjects. Second, quantitative courses do not usually provide immediate solutions to real problems but at best provide a mediated solution, something that students in the first years are likely to perceive negatively. Nevertheless, it is necessary to highlight that the “gratification effect” is a purely speculative hypothesis, as it is possible that confounding factors account for part (or much) of this effect.

Regardless of the causes, from our point of view, the results obtained make it necessary to reconsider the use of SETs in many Spanish universities, where it is relatively common to compare evaluations in all areas.

The main limitation of this article is that we have considered only one university institution and only the business and law school. For this reason, we propose a future line of research that replicates this work by considering a more diverse sample. However, since the results are very similar to those of Uttl & Smibert (2017), even with the considerable differences in the samples used, we believe that very similar results will most likely be obtained. Therefore, in our opinion, the findings obtained enjoy considerable robustness. A second limitation is that we have not controlled for perceived hardness, workload needed, or other variables that may act as confounding factors that could potentially account for the differential pattern between undergraduate and master courses. However, this limitation does not invalidate the main conclusion of this article; regardless of the causes (higher perceived hardness, higher workload needed, or simply that students do not like mathematics), it is clear that in undergraduate courses, there exists a considerable negative bias in SETs, which is detrimental to quantitative courses.

## Conclusions

Throughout this article, we analyzed SETs at a midsize Spanish university from 2016/2017–2019/2020. All the data included in this analysis were obtained from official university surveys developed by a team of experts in teaching quality. The sample for the undergraduate courses consists of 80,667 SETs and 2,885 classes in which 488 instructors and 322 different courses were evaluated. The sample for the master’s courses consists of 16,083 SETs, 871 classes, 275 instructors, and 155 different courses. The results show that in the case of undergraduate courses, there is a considerable difference between courses that use quantitative methods and other areas. The relative risk rates, regardless of the cutoff criterion used to distinguish between “good performance” and “bad performance”, are clearly unfavorable toward instructors of quantitative courses, who are much more likely to be classified as “bad teachers”. While the differences depend on the area against which the comparison is made, in all cases, the comparison is unfavorable. Most interesting is the consistency with Uttl & Smibert’s (2017) analysis, as strikingly similar results have been obtained even with considerable differences between the samples used. However, in the case of master’s courses, the situation is entirely different, and no significant differences between quantitative courses and the remaining courses are apparent, except for some cutoff criteria.

It seems clear that quantitative courses are not appreciated by students, at least in undergraduate courses in business and law school. This is probably due to a problem that goes back to high school. In this sense, perhaps more effort should be made in preuniversity studies to make this type of course more attractive by giving the courses greater proximity to real problems and using a more practical approach.

These results lead to three different conclusions. First, the hypothesis that the marked negative bias toward quantitative courses in undergraduate courses is due to the instructors’ deficits seems to be unjustified. Our results suggest that the same professor is evaluated very differently depending on whether he or she teaches undergraduate or master’s degree courses. Second, it is essential to avoid comparing SETs between different areas of knowledge, at least at the undergraduate level. The results clearly show a strong negative bias toward professors who teach quantitative courses, so any comparison with the SETs obtained by professors in other areas of knowledge is clearly unfair. The most obvious example is found in Table 5. If we use a cutoff criterion equal to the average SET in all subjects, 74.9% of quantitative methods instructors will be included in the “bad performance” category. However, only 30.9% of language instructors fall into this category. This leads us to the third conclusion, namely, if there is no significant change in the use and interpretation of SETs, an adverse selection process may occur. Cashin (1990) argued that many of the low-rated fields require institutions to pay very high salaries to compete with business and industry. In other words, only the worst professionals opted for teaching since the good professionals had better alternatives in the industry, which meant that teachers were worse in these areas than in other areas. Our results suggest that this is not the case. However, this is a substantial risk for the future. Currently, a specialist in quantitative methods has more economically attractive alternatives in the industry than in universities, at least in the case of Spain. After 5 years of doctoral studies, the best option in a university is a position as an “Ayudante Doctor” (the lowest level in the Spanish system), which implies a salary of between 25,000 and 28,000 euros per year, depending on the university. This salary is practically the same as what a graduate in science without any additional studies can obtain in the corporate world immediately after finishing his or her degree. Furthermore, the salary is undoubtedly much lower than the salary that he or she will obtain after 5 years. Therefore, if he or she chooses to develop his or her professional career in academia, it is because of a strong vocational calling. However, if he or she finds himself/herself in an environment in which he or she obtains systematically lower SETs than his or her peers in other areas, which results in lower possibilities of receiving tenure, promotion, or merit pay, he or she may reconsider the decision. This would lead to Cashin’s statement becoming true; in the long run, it is possible that only professionals who are not able to obtain a better job in the industry will opt for academia. If there are no changes in the use and interpretation of SETs in a few years, we may see that the differences between the SETs received by instructors of quantitative methods and those of other areas are even greater. Then, there will be an objective reason for this.

From a more general perspective, concerning the validity of the SETs, this work proves that the area of knowledge generates a considerable bias in SETs. Therefore, the practice of comparing SETs between different areas of knowledge should be discontinued immediately. One possibility to correct this bias is to establish a within-field percentile of the rating average. The cutoff criterion to distinguish good and bad teachers would be the percentile determined by the university. In this way, the comparison would be carried out exclusively between instructors in the same area. It would also be possible to calculate the within-field z-score for each professor; the number of standard deviations a given instructor lies above or below the mean would in this case be the indicator to use. Unlike what happens with other noninstructional biases such as accent bias or gender bias, in this case, the solution seems feasible and depends only on a political decision, that is, on the way SETs are used. However, another problem arises; countless previous studies have shown that SETs depend on multiple factors unrelated to professors’ effectiveness. Therefore, to make SETs reliable instruments, it would be necessary to correct these biases as well. This is, for example, the proposal by Berezvai, Lukáts & Molontay (2021: 806) to correct the bias derived from GPA (teachers who award higher grades get better SETs): “A potential way to obtain an unbiased SET score would be to subtract 0.3 or 0.4 multiplied by the difference in course grade average compared to the average grade of all students in the entire faculty”. This approach would lead to the need to correct all biases similarly. Then, a third problem emerges; given the results of previous research, it seems that both noninstructional biases and their magnitude may vary significantly even from one class to another, depending on the characteristics of the learners. Thus, the correction rules would have to be specific to virtually every class, which is not possible. Consequently, we are skeptical about the possibility of correcting the results of SETs to make them valid instruments. We think it is possible to mitigate noninstructional biases, and a good start would be to eliminate comparisons between different areas of knowledge, but it does not seem possible to eliminate biases completely. SETs can perhaps be used to identify extreme cases (very high or very low performers, with similar behavior over a long period). However, even in these cases, the results should be taken with caution, aware that there is no certainty that these results are indicators of better teaching effectiveness but that they may be due to the sum of multiple unrelated factors. In conclusion, alternative mechanisms for faculty evaluation should be sought. Moreover, those universities that, despite all the problems indicated above, decide to continue using SETs should at least use them in a very different way.

## Supplemental Information

10.7717/peerj.13456/supp-1 Supplemental Information 1 SETs for degree and master courses.

## Additional Information and Declarations

### Data Availability

The following information was supplied regarding data availability: The raw measurements are available in the Supplemental File.

## References

[ref-1] Anderson-Hsieh J, Koehler K (1988). The effect of foreign accent and speaking rate on native speaker comprehension. Language Learning.

[ref-3] Arroyo-Barrigüete JL, Obregón García A, Rua Vieites A, Ortiz Lozano JM (2021). Impact of noninstructional factors on undergraduate student evaluation of teaching. http://hdl.handle.net/11531/66701.

[ref-2] Auguie B (2017). gridExtra: miscellaneous functions for “Grid” Graphics. https://CRAN.R-project.org/package=gridExtra.

[ref-4] Benjamin DJ, Berger JO, Johannesson M, Nosek BA, Wagenmakers EJ, Berk R, Bollen KA, Börn Brembs, Brown L, Camerer C, Cesarini D, Chambers CD, Clyde M, Cook TD, De Boeck P, Dienes Z, Dreber A, Easwaran K, Efferson C, Fehr E, Fidler F, Field AP, Forster M, George EI, Gonzalez R, Goodman S, Green E, Green DP, Greenwald AG, Hadfield JD, Hedges LV, Held L, Hua Ho T, Hoijtink H, Hruschka DJ, Imai K, Imbens G, Ioannidis JPA, Jeon M, Jones JH, Kirchler M, Laibson D, List J, Little R, Lupia A, Machery E, Maxwell SE, McCarthy M, Moore DA, Morgan SL, Munafó M, Nakagawa S, Nyhan B, Parker TH, Pericchi L, Perugini M, Rouder J, Rousseau J, Savalei V, Schönbrodt FD, Sellke T, Sinclair B, Tingley D, Van Zandt T, Vazire S, Watts DJ, Winship C, Wolpert RL, Xie Y, Young C, Zinman J, Johnson VE (2018). Redefine statistical significance. Nature Human Behaviour.

[ref-5] Beran T, Violato C (2005). Ratings of university teacher instruction: how much do student and course characteristics really matter?. Assessment & Evaluation in Higher Education.

[ref-6] Berezvai Z, Lukáts GD, Molontay R (2021). Can professors buy better evaluation with lenient grading? The effect of grade inflation on student evaluation of teaching. Assessment & Evaluation in Higher Education.

[ref-7] Braga M, Paccagnella M, Pellizzari M (2014). Evaluating students’ evaluations of professors. Economics of Education Review.

[ref-8] Carrell SE, West JE (2010). Does professor quality matter? Evidence from random assignment of students to professors. Journal of Political Economy.

[ref-9] Cashin WE (1990). Students do rate different academic fields differently. New Directions for Teaching and Learning.

[ref-10] Centra JA (2009). Differences in responses to the student instructional report: is it bias.

[ref-11] Clayson DE (2009). Student evaluations of teaching: are they related to what students learn? A meta-analysis and review of the literature. Journal of Marketing Education.

[ref-12] Clayson D (2022). The student evaluation of teaching and likability: what the evaluations actually measure. Assessment & Evaluation in Higher Education.

[ref-13] DeFrain E (2016). An analysis of differences in non-instructional factors affecting teacher-course evaluations over time and across disciplines.

[ref-14] Felton J, Mitchell J, Stinson M (2004). Web-based student evaluations of professors: the relations between perceived quality, easiness and sexiness. Assessment & Evaluation in Higher Education.

[ref-15] Felton J, Koper PT, Mitchell J, Stinson M (2008). Attractiveness, easiness and other issues: Student evaluations of professors on ratemyprofessors.com. Assessment & Evaluation in Higher Education.

[ref-16] Fox J (2020). RcmdrMisc: R commander miscellaneous functions. https://CRAN.R-project.org/package=RcmdrMisc.

[ref-17] Goldin-Meadow S, Kim S, Singer M (1999). What the teacher’s hands tell the student’s mind about math. Journal of Educational Psychology.

[ref-18] Hornstein HA (2017). Student evaluations of teaching are an inadequate assessment tool for evaluating faculty performance. Cogent Education.

[ref-19] Hoyt DP, Lee E (2002). Technical Report No. 12: basic data for the revised IDEA system. The IDEA Center. https://cutt.ly/9OqcNi3.

[ref-20] Kornell N, Hausman H (2016). Do the best teachers get the best ratings?. Frontiers in Psychology.

[ref-21] La Rocca M, Parrella ML, Primerano I, Sulis I, Vitale MP (2017). An integrated strategy for the analysis of student evaluation of teaching: from descriptive measures to explanatory models. Quality & Quantity.

[ref-22] Nakazawa M (2019). fmsb: functions for medical statistics book with some demographic data. https://CRAN.R-project.org/package=fmsb.

[ref-23] OECD (2018). PISA results. https://www.oecd.org/pisa/publications/.

[ref-24] Pedrosa C (2020). Actitudes hacia las matemáticas en estudiantes universitarios.

[ref-46] R Core Team (2020). R: a language and environment for statistical computing. https://www.R-project.org/.

[ref-25] Rampichini C, Grilli L, Petrucci A (2004). Analysis of university course evaluations: from descriptive measures to multilevel models. Statistical Methods and Applications.

[ref-26] Rosen AS (2018). Correlations, trends and potential biases among publicly accessible web-based student evaluations of teaching: a large-scale study of RateMyProfessors.com data. Assessment & Evaluation in Higher Education.

[ref-27] Royal KD, Stockdale MR (2015). Are teacher course evaluations biased against faculty that teach quantitative methods courses?. International Journal of Higher Education.

[ref-28] Sanchez CA, Khan S (2016). Instructor accents in online education and their effect on learning and attitudes. Journal of Computer Assisted Learning.

[ref-29] Scholz F, Zhu A (2019). kSamples: K-sample rank tests and their combinations. https://CRAN.R-project.org/package=kSamples.

[ref-30] Stroebe W (2020). Student evaluations of teaching encourages poor teaching and contributes to grade inflation: a theoretical and empirical analysis. Basic and Applied Social Psychology.

[ref-31] Sulis I, Porcu M, Capursi V (2019). On the use of student evaluation of teaching: a longitudinal analysis combining measurement issues and implications of the exercise. Social Indicators Research.

[ref-32] Stonebraker RJ, Stone GS (2015). Too old to teach? The effect of age on college and university professors. Research in Higher Education.

[ref-33] Tran TTT, Do TX (2020). Student evaluation of teaching: do teacher age, seniority, gender, and qualification matter?. Educational Studies.

[ref-34] Uttl B, White CA, Morin A (2013). The numbers tell it all: students don’t like numbers!. PLOS ONE.

[ref-35] Uttl B, Smibert D (2017). Student evaluations of teaching: teaching quantitative courses can be hazardous to one’s career. PeerJ.

[ref-36] Uttl B, White CA, Gonzalez DW (2017). Meta-analysis of faculty’s teaching effectiveness: student evaluation of teaching ratings and student learning are not related. Studies in Educational Evaluation.

[ref-37] Uttl B, Bell S, Banks K (2018). Student evaluation of teaching (SET) ratings depend on the class size: a systematic review.

[ref-38] Uttl B, Rollett W, Bijlsma H, Röhl S (2021). Lessons learned from research on student evaluation of teaching in higher education. Student Feedback on Teaching in Schools.

[ref-39] Uttl B, Violo VC (2021). Small samples, unreasonable generalizations, and outliers: gender bias in student evaluation of teaching or three unhappy students?. ScienceOpen Research.

[ref-40] Wallisch P, Cachia J (2019). Determinants of perceived teaching quality: the role of divergent interpretations of expectations. https://psyarxiv.com/dsvgq/.

[ref-41] Wickham H (2016). ggplot2: elegant graphics for data analysis.

[ref-42] Wickham H, Bryan J (2019). readxl: read Excel files. https://CRAN.R-project.org/package=readxl.

[ref-43] Wickham H, François R, Henry L, Müller K (2020). dplyr: a grammar of data manipulation. https://CRAN.R-project.org/package=dplyr.

[ref-44] Wolbring T (2012). Class attendance and students’ evaluations of teaching: do no-shows bias course ratings and rankings?. Evaluation Review.

[ref-45] Yunker PJ, Yunker JA (2003). Are student evaluations of teaching valid? Evidence from an analytical business core course. Journal of Education for Business.

